# P60 Antimicrobial resistance among female patients with uncomplicated urinary tract infections in England: a physician panel chart review

**DOI:** 10.1093/jacamr/dlaf118.067

**Published:** 2025-07-14

**Authors:** Mary E Georgiou, Dina Lad, Matthew Helgeson, Vanessa Cortes, Philip Morgan, Ashish V Joshi, Madison T Preib, Tracy Guo, Daisy Liu, Ellen Sears, Maryaline Catillon, Rose Chang, Mei Sheng Duh, Fanny S Mitrani-Gold

**Affiliations:** GSK, London, UK; GSK, London, UK; GSK, Collegeville, PA, USA; GSK, Bogota, Colombia; GSK, London, UK; GSK, Collegeville, PA, USA; GSK, Collegeville, PA, USA; Analysis Group Inc., Boston, MA, USA; Analysis Group Inc., Boston, MA, USA; Analysis Group Inc., New York, NY, USA; Analysis Group Inc., New York, NY, USA; Analysis Group Inc., Boston, MA, USA; Analysis Group Inc., New York, NY, USA; GSK, Collegeville, PA, USA

## Abstract

**Background:**

Antimicrobial resistance (AMR) is a significant and growing public health concern. Data on the prevalence of AMR can optimize antimicrobial prescribing choices and highlight unmet need in the treatment landscape. Surveillance data for uncomplicated urinary tract infection (uUTI)—a leading reason for antibiotic prescription—are lacking. This study assessed the prevalence of AMR among patients with uUTI in England.

**Methods:**

This retrospective physician panel chart review was conducted between 1 July 2020 and 30 June 2022. Primary endpoints were prevalence of AMR overall and proportion of patients in specified resistance sub-cohorts (with isolates susceptible [SUS], resistant to 1–2 drug classes [DR 1–2], or resistant to ≥3 drug classes [multidrug resistant, or MDR3+]). Females aged ≥18 years, with an outpatient diagnosis of uUTI (confirmed via a urine culture positive for *Escherichia coli*) and susceptibility test results for ≥3 drug classes, were eligible. Prevalence of AMR was assessed ±5 days around the index date (defined as the first diagnosis of uUTI within the study period). Demographic and clinical characteristics were assessed during the 12-month baseline period prior to or on the index date.

**Results:**

Overall, 461 eligible patients were recruited from general practitioners (78.9%), urologists (14.9%) and infectious disease specialists (6.2%). Mean age (SD) was 40.7 (16.4) years and most patients (58.6%) were from Southern England. Patients’ history included: uUTI (34.1%); recurrent uUTI (≥2 uUTIs in 6 months or ≥3 uUTIs in 12 months; 20.0%); AMR (8.0%). Susceptibility testing frequency varied by antibiotic (nitrofurantoin [100%]; trimethoprim [98.5%]; pivmecillinam [56.6%]; fosfomycin [39.3%]; amoxicillin/co-amoxiclav [90.9%]; cefalexin [64.2%]; fluoroquinolones [36.2%]) and broadly reflected treatment guidelines. AMR prevalence was: nitrofurantoin (38/461, 8.2%); trimethoprim (124/454, 27.3%); pivmecillinam (14/261, 5.4%); fosfomycin (11/181, 6.1%); amoxicillin/co-amoxiclav (120/419, 28.6%); cefalexin (37/296, 12.5%); fluoroquinolones (23/167, 13.8%). The proportions of patients in resistance sub-cohorts were: SUS (51.8%, *n*=239); DR 1–2 (43.6%, *n*=201); MDR3+ (4.6%, *n*=21). Among patients categorized as MDR3+, prevalence of AMR was 10/21 (47.6%) for nitrofurantoin and 16/21 (76.2%) for trimethoprim.

**Conclusions:**

Almost half (48.2%) of patients with *E. coli* UTIs in England had isolates resistant to ≥1 drug class; of which 4.6% had isolates resistant to ≥3 classes. AMR to first-line therapies was low overall for nitrofurantoin (8.2%) and moderate to high for trimethoprim (27.3%). Rates of resistance among patients categorized as MDR3+ were high (nitrofurantoin, 47.6%; trimethoprim, 76.2%). This suggests that new treatment options could benefit patients infected with MDR *E. coli*. A lack of comprehensive susceptibility testing for some antibiotics may cause the true prevalence of AMR in uUTI to be underestimated.

